# Machine learning algorithm to predict postoperative bleeding complications after lateral decubitus percutaneous nephrolithotomy

**DOI:** 10.1097/MD.0000000000037050

**Published:** 2024-01-26

**Authors:** Rui Meng, Weining Wang, Zhipeng Zhai, Chao Zuo

**Affiliations:** a Department of Urology, YuQuan Hospital, Tsinghua University, Beijing, China; b Department of Urology, Peking University First Hospital - Miyun Hospital, Beijing, China.

**Keywords:** bleeding, kidney stones, machine learning, percutaneous nephrolithotomy, random forest

## Abstract

Bleeding is a serious complication following percutaneous nephrolithotomy (PCNL). This study establishes a predictive model based on machine learning algorithms to forecast the occurrence of postoperative bleeding complications in patients with renal and upper ureteral stones undergoing lateral decubitus PCNL. We retrospectively collected data from 356 patients with renal stones and upper ureteral stones who underwent lateral decubitus PCNL in the Department of Urology at Peking University First Hospital-Miyun Hospital, between January 2015 and August 2022. Among them, 290 patients had complete baseline data. The data was randomly divided into a training group (n = 232) and a test group (n = 58) in an 8:2 ratio. Predictive models were constructed using Logistic Regression, Random Forest, and Extreme Gradient Boosting (XGBoost). The performance of each model was evaluated using Accuracy, Precision, F1-Score, Receiver Operating Characteristic curves, and Area Under the Curve (AUC). Among the 290 patients, 35 (12.07%) experienced postoperative bleeding complications after lateral decubitus PCNL. Using postoperative bleeding as the outcome, the Logistic model achieved an accuracy of 73.2%, AUC of 0.605, and F1 score of 0.732. The Random Forest model achieved an accuracy of 74.5%, AUC of 0.679, and F1 score of 0.732. The XGBoost model achieved an accuracy of 68.3%, AUC of 0.513, and F1 score of 0.644. The predictive model for postoperative bleeding after lateral decubitus PCNL, established based on machine learning algorithms, is reasonably accurate. It can be utilized to predict postoperative stone residue and recurrence, aiding urologists in making appropriate treatment decisions.

## 1. Introduction

Kidney stones are prevalent in almost all regions of the world, with increasing incidence reported in multiple areas.^[1–3]^ They can lead to urinary tract infections, urinary obstruction, hematuria, and impaired renal function.^[4,5]^ Without timely or conservative treatment, kidney stones can progress to renal failure or even death.^[6]^ Therefore, active intervention is essential for managing kidney stones. Common treatment modalities for stones include Extracorporeal Shock Wave Lithotripsy, Ureteroscopy, Percutaneous Nephrolithotomy (PCNL), and occasionally open surgery.^[7]^ PCNL is the preferred surgical method for treating large, multiple, and complex kidney stones.^[8–10]^

Despite PCNL being a mature surgical technique in recent years, complications remain common, with approximately 23.3% of patients experiencing postoperative complications.^[11]^ Bleeding is a serious complication that requires prompt control and management. While conservative methods are often sufficient to control most post-PCNL bleeding, a subset of patients with severe bleeding may require surgical interventions such as angiographic embolization.^[12]^ Moreover, previous studies have found that postoperative bleeding can lead to transfusions, with transfusion rates reaching up to 55%.^[13]^ Therefore, the establishment of a predictive model for post-PCNL bleeding holds clinical significance, providing guidance for clinicians in diagnosis and treatment.

Machine Learning methods, developed from branches of statistics, computer science, and artificial intelligence, enable us to uncover complex relationships and patterns in large databases that classical statistical methods may not detect, resulting in improved and more useful predictive models.^[14,15]^ In recent years, machine learning predictive models for urological diseases have been widely reported.^[16,17]^ However, there is currently no reported machine learning predictive model for postoperative bleeding after lateral decubitus PCNL. This study aims to construct a machine learning predictive model for complications related to postoperative bleeding after lateral decubitus PCNL.

## 2. Materials and methods

### 2.1. Study population

We retrospectively collected data from 356 patients with renal stones and upper ureteral stones who underwent lateral decubitus PCNL in the Department of Urology at Peking University First Hospital-Miyun Hospital, between January 2015 and August 2022. Among them, 290 patients had complete baseline data. Using 26 clinical data parameters such as gender, age, and stone size as variables, we constructed a machine learning predictive model with postoperative bleeding as the outcome.

In this study, the endpoint of the research was postoperative bleeding, defined as a decrease in hemoglobin greater than or equal to 20 g/L between preoperative and the first day postoperative. A decrease of <20 g/L was not considered a complication of postoperative bleeding. The formula for calculating the decrease in hemoglobin (g/L) is: Decrease = Preoperative hemoglobin - Hemoglobin on the first postoperative day.

Patients were included based on the following criteria: Confirmation of the presence of renal or upper ureteral stones through ultrasound, intravenous pyelography, or urologic system CT before surgery; Lateral decubitus position during PCNL; Patients with complete clinical data.

Exclusion criteria included: Patients with abnormal coagulation function; Patients with cardiorespiratory dysfunction unable to undergo surgery; Patients unable to cooperate with the study.

**Figure 1.**
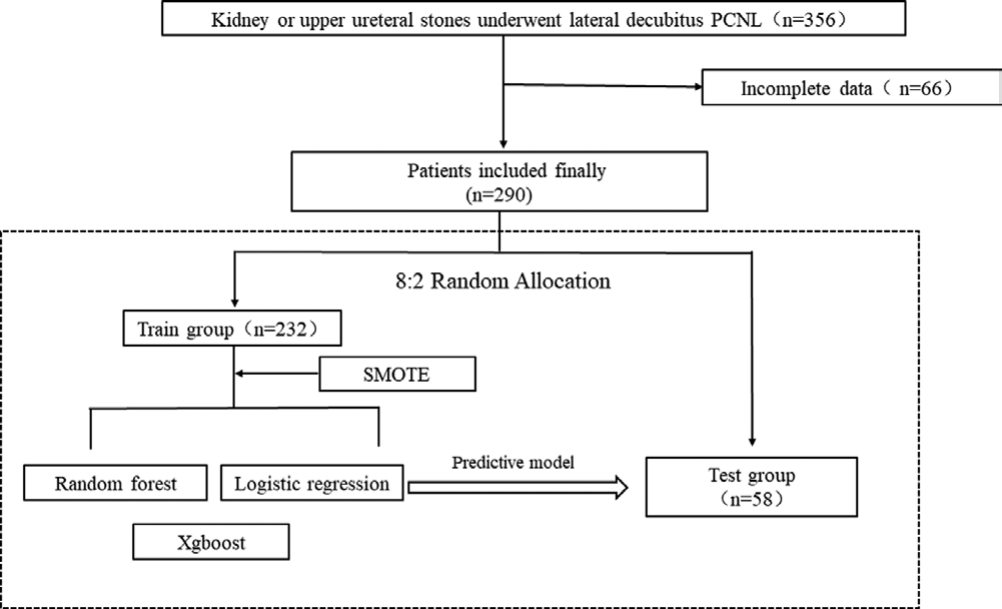
Flowchart of the study.

Patients meeting the inclusion criteria were randomly divided into a training group (n = 232) and a test group (n = 58) in an 8:2 ratio. The Synthetic Minority Over-sampling Technique algorithm was applied to augment the dataset, addressing data imbalance issues.^[18]^ A machine learning algorithm was employed to construct a predictive model for postoperative bleeding after lateral decubitus PCNL using the training group, and the accuracy of the model was tested using the test group. The process flow is illustrated in Figure 1.

This study was conducted in accordance with the principles of the Helsinki Declaration (2013 revised edition) and received approval from the Ethics Committee of Peking University First Hospital-Miyun Hospital. Informed consent for this retrospective analysis was waived by the committee.

### 2.2. Study variables

The variables included in the model construction for this study are as follows: Gender, Age, BMI, Hypertension, Diabetes mellitus, Coronary Heart Disease, Lung Diseases, Brain Diseases, spinal deformity, Lesion side, Stone location, Stone size, Multiple stones, Puncture site, Channel type, Number of channels, Operation time, Intraoperative blood loss, Preoperative systolic blood pressure (SBP), Preoperative diastolic blood pressure (DBP), Intraoperative SBP, Intraoperative DBP, Preoperative heart rate, Intraoperative heart rate, Mode of anesthesia, Stone co-infection.

### 2.3. Machine learning analysis

**Figure 2.**
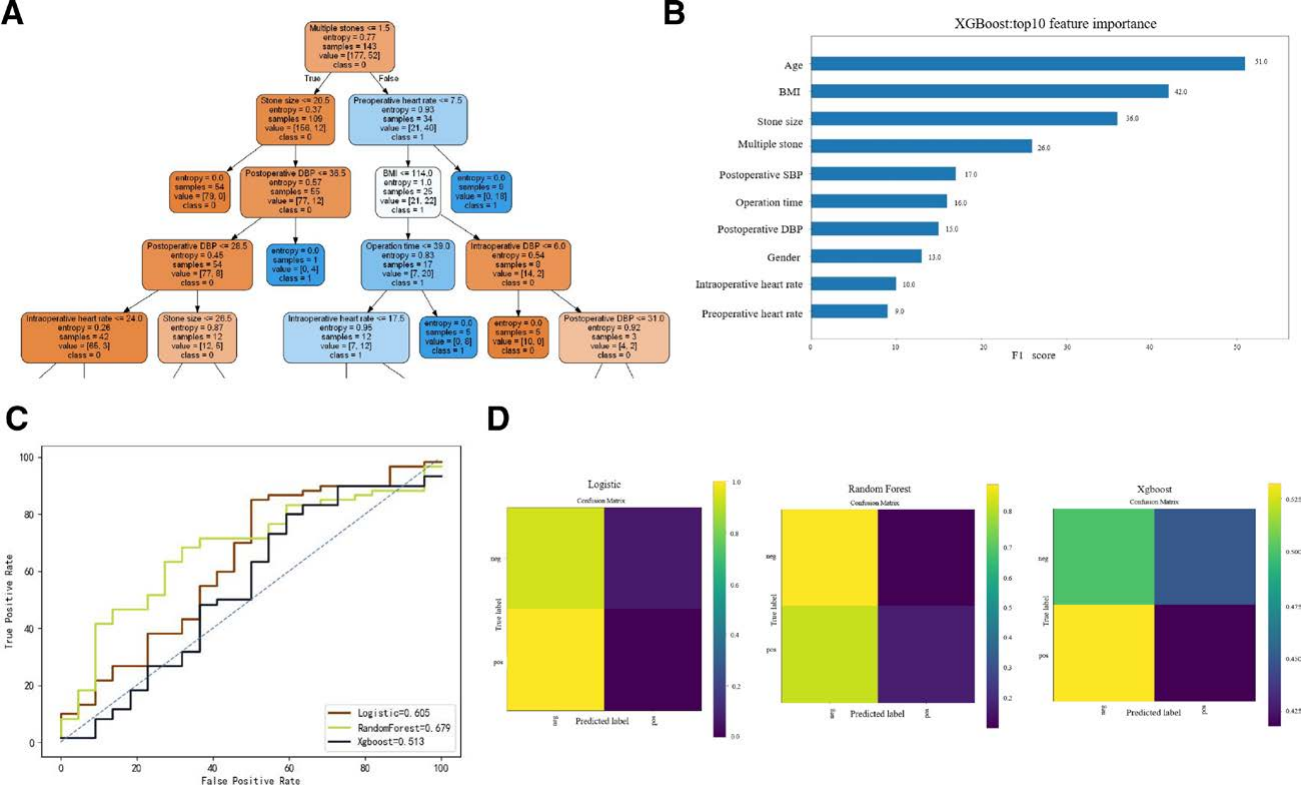
(A) It is visualization in parts of leaves based on the Random forest model. It should be noted that the visual model only shows a part of the leaves or principles of the decision tree and does not represent the entire model. (B) Top 10 important features of XGBoost model. (C) Typical receiver operating characteristic curve in 3 models. (D) Confusion matrix of 3 models. XGBoost = extreme gradient boosting.

Three models were constructed, including the Logistic Regression model, Random Forest model, and Extreme Gradient Boosting (XGBoost) model. Logistic Regression is a traditional model commonly used in many studies. Random Forest is an ensemble learning algorithm designed for predicting binary outcomes (classifiers). It creates a decision tree forest through bootstrap aggregation of samples and features. Random Forest can easily assess the importance or contribution of variables to the model.^[19,20]^ The XGBoost model employs classification trees as weak learners, learning a binary logistic objective function. The boosting method redefines weak classifiers (decision trees) iteratively as residuals of the previous model, achieving higher predictive accuracy through multiple iterations and forming a strong classifier.^[21]^ Figure 2A to B illustrate the parameters of the Random Forest and XGBoost models, respectively, showcasing some processes of the Random Forest model and the top ten most important variables of the XGBoost model. The performance of each predictive model was evaluated using Accuracy, Precision, F1 score, Receiver Operating Characteristic curves, and Area Under the Curve (AUC).

### 2.4. Statistical analysis

Statistical analysis was performed using SPSS version 22.0. Normally distributed continuous data are expressed as mean ± standard deviation, while skewed data are described using median (range). For continuous variables, t-tests were used for normally distributed variables, and the Mann–Whitney U test was employed for non-normally distributed variables. Chi-square tests and Fisher exact probability method were used to analyze categorical variables.

## 3. Results

### 3.1. Patient characteristics

The baseline characteristics of the patients are presented in Table 1. The average age of the patients was 51.72 ± 13.11 years. Of the total, 179 (61.72%) were male, and 111 (38.28%) were female. The mean stone size was 2.86 ± 0.95 cm. The average surgical duration was 115.31 ± 49.67 minutes. The mean intraoperative blood loss was 19.25 ± 22.19 ml. Thirty-five patients experienced postoperative bleeding complications, resulting in an incidence rate of 12.07%.

**Table 1:** Basic characteristics of the patients.

Variable	Mean (SD) or n/N
Patients	290
Mean age (yr)	51.72 ± 13.11
BMI (kg/m^2^)	25.54 ± 4.04
Gender, n (%)	
Male	179 (61.72)
Female	111 (38.28)
Hypertension, n (%)	
Yes	92 (31.72)
No	198 (68.28)
Diabetes mellitus, n (%)	
Yes	51 (17.59)
No	239 (82.51)
CHD, n (%)	
Yes	19 (6.55)
No	271 (93.45)
Lung diseases, n (%)	
Yes	0 (0.00)
No	290 (100.00)
Brain diseases, n (%)	
Yes	14 (4.83)
No	276 (95.17)
Spinal deformity, n (%)	
Yes	5 (1.72)
No	285 (98.18)
Lesion side, n (%)	
Unilateral	243 (83.79)
Bilateral	47 (16.21)
Stone location, n (%)	
Kidney/ureteral stones	255 (87.93)
Kidney and ureteral stones	35 (12.07)
Multiple stones, n (%)	
Yes	69 (23.79)
No	221 (76.21)
Puncture site, n (%)	
Upper/lower renal calices	44 (15.17)
Median renal calices	246 (84.83)
Channel type, n (%)	
Standard channel	54 (18.62)
Microchannel	236 (81.38)
Number of channels (unit)	1.04 ± 0.19
Stone size (cm)	2.86 ± 0.95
Operation time (min)	115.31 ± 49.67
Intraoperative blood loss (ml)	19.25 ± 22.19
Preoperative SBP (mm Hg)	132 ± 17.47
Preoperative DBP (mm Hg)	82 ± 10.77
Intraoperative SBP (mm Hg)	119 ± 14.57
Intraoperative DBP (mm Hg)	67 ± 12.08
Preoperative heart rate (bpm)	75.54 ± 9.54
Intraoperative heart rate (bpm)	65.20 ± 10.58
Stone co-infection, n (%)	
Yes	97 (33.45)
No	193 (66.55)
Mode of anesthesia, n (%)	
General anesthesia	270 (93.10)
Combined epidural anesthesia	20 (6.90)
Postoperative hemorrhage, n (%)	
Yes	35 (12.07)
No	255 (87.93)

BMI = body mass index, bpm = beats per minute, CHD = coronary artery heart disease, DBP = diastolic blood pressure, SBP = systolic blood pressure.

### 3.2. Efficiency of machine learning models in predicting postoperative bleeding after lateral decubitus PCNL

The 290 patients were randomly divided into 2 datasets, with 80% assigned to the training group (n = 232) and 20% to the test group (n = 58). There were no statistically significant differences in various indicators between the 2 groups (*P* > .05) (Table 2). The accuracy of the Logistic model was 73.2%, with an AUC of 0.605 and an F1 score of 0.732. The Random Forest model achieved an accuracy of 74.5%, an AUC of 0.679, and an F1 score of 0.732. The XGBoost model showed an accuracy of 68.3%, an AUC of 0.513, and an F1 score of 0.644 (Fig. 2C and Table 3). The generated confusion matrix indicated that the Random Forest model had the best predictive performance among all models (Fig. 2D).

**Table 2:** Basic characteristics of the patients of postoperative hemorrhage.

Variable	Train group	Test group	*P* value
Patients	232	58	
Mean age (yr)	51.06 ± 12.44	54.40 ± 15.32	.084
BMI (kg/m^2^)	25.61 ± 3.92	25.27 ± 4.52	.576
Gender, n (%)			.205
Male	139 (59.9)	40 (69.0)	
Female	93 (41.1)	18 (31.0)	
Hypertension, n (%)			.089
Yes	79 (34.1)	13 (22.4)	
No	153 (65.9)	45 (77.6)	
Diabetes mellitus, n (%)			.105
Yes	45 (19.4)	6 (10.3)	
No	187 (81.6)	52 (897)	
CHD, n (%)			.55
Yes	15 (6.5)	4 (6.9)	
No	217 (93.5)	54 (93.1)	
Lung diseases n (%)			
Yes	0 (0)	0 (0)	1.000
No	232 (100)	58 (100)	
Brain diseases n (%)			.891
Yes	11 (4.7)	3 (5.2)	
No	221 (95.3)	55 (94.8)	
Spinal deformity n (%)			1.000
Yes	4 (1.7)	1 (1.7)	
No	228 (98.3)	57 (98.3)	
Preoperative SBP (mm Hg)	133.58 ± 16.92	94.50 ± 3.5	.167
Preoperative DBP (mm Hg)	82.46 ± 9.29	71.50 ± 8.5	.200
Intraoperative SBP (mm Hg)	119.19 ± 14.98	90.00 ± 0.00	.452
Intraoperative DBP (mm Hg)	54.44 ± 4.97	50.00 ± 0.00	.541
Preoperative heart rate (bpm)	65.76 ± 10.96	62.74 ± 8.41	.079
Intraoperative heart rate (bpm)	75.98 ± 9.69	75.26 ± 9.01	.613
Lesion side, n (%)			.146
Unilateral	196 (84.5)	47 (81.0)	
Bilateral	36 (15.5)	11 (19.0)	
Stone location, n (%)			.774
Kidney/ureteral stones	205 (92.7)	50 (69.0)	
Kidney and ureteral stones	37 (7.3)	8 (31.0)	
Multiple stones, n (%)			.535
Yes	57 (24.6)	12 (20.7)	
No	175 (75.4)	46 (79.3)	
Puncture site, n (%)			.439
Upper/lower renal calices	36 (15.5)	8 (13.8)	
Median renal calices	196 (84.5)	50 (86.2)	
Channel type, n (%)			.407
Standard channel	41 (17.7)	13 (22.4)	
Microchannel	191 (82.3)	45 (77.6)	
Number of channels (unit)	1.07 ± 0.15	1.06 ± 0.31	.397
Stone size (cm)	2.96 ± 0.96	3.04 ± 0.82	.531
Operation time (min)	121.38 ± 40.18	120.97 ± 72.80	.701
Intraoperative blood loss (mL)	18.62 ± 22.26	21.76 ± 21.92	.339
Stone co-infection, n (%)			.081
Yes	42 (18.1)	14 (24.1)	
No	190 (81.9)	34 (75.9)	
Mode of anesthesia, n (%)			.247
General anesthesia	218 (94.0)	52 (89.7)	
Combined epidural anesthesia	14 (6.0)	6 (10.3)	
Postoperative hemorrhage, n (%)			.367
Yes	26 (11.2)	9 (15.5)	
No	206 (88.8)	49 (84.5)	

CHD = coronary heart disease, DBP = diastolic blood pressure, SBP = systolic blood pressure.

**Table 3:** Performance of each model of the postoperative hemorrhage prediction model.

Models	Accuracy		Precision		AUC		F1 score	
	Average	95% CI	Average	95% CI	Average	95% CI	Average	95% CI
Logistic regression	0.732	0.690–0.774	0.72	0.698–0.742	0.605	0.573–0.637	0.732	0.705–0.759
Random forest	0.745	0.713–0.777	0.733	0.703–0.763	0.679	0.627–0.731	0.732	0.690–0.774
Xgboost	0.683	0.656–0.710	0.659	0.617–0.701	0.513	0.483–0.543	0.644	0.612–0.676

AUC = area under the curve, XGBoost = extreme gradient boosting.

## 4. Discussion

PCNL is a safe and effective surgical procedure used for the removal of large, complex, and multiple kidney stones.^[22]^ However, life-threatening bleeding complications may occur during and after PCNL. Adequate treatment options for bleeding resulting from PCNL, such as placing larger nephrostomy tubes, clamping the nephrostomy tube, balloon tamponade, and vascular embolization, have demonstrated good efficacy.^[23,24]^ Early intervention with treatments like placing larger fistula tubes in high-risk patients can effectively prevent postoperative bleeding. Therefore, a reliable postoperative bleeding prediction model is beneficial for clinicians in making diagnostic and therapeutic decisions.

Previous research primarily focused on identifying factors influencing postoperative bleeding, providing references for clinicians to identify high-risk patients prone to postoperative bleeding. In the study by Tolga Akman et al,^[25]^ diabetes, surgical time, number of surgeries, and stone type were found to be correlated with a decrease in hemoglobin levels. Srivastava et al^[26]^ suggested that stone size is the sole important factor predicting post-PCNL bleeding. In the research by Jeong Kuk Lee et al,^[27]^ BMI, stone size, stone location, surgical time, and preoperative renal pelvic dilation were identified as predictive factors for severe bleeding during PCNL. In our study, the Random Forest model identified multiple stones, stone size, preoperative heart rate, BMI, and intraoperative DBP as the top 5 influential variables. The XGBoost model highlighted age, BMI, stone size, multiple stones, and intraoperative SBP as the top 5 important features. Stone size, multiple stones, and BMI as significant features align well with previous studies and are close to clinical reality.

Currently, there is only one report on predicting post-PCNL bleeding through multivariate regression analysis using traditional statistical methods. This study represents the first application of a machine learning-based predictive model for post-PCNL bleeding. Traditional statistical methods, such as regression analysis, may not extract features from data as effectively as machine learning algorithms, resulting in a potential disadvantage in model construction with the same sample size. Giorgio Mazzon et al‘s study,^[28]^ a prospective large-scale population study with 1980 patients, identified factors such as age (*P* = .041), BMI (*P* = .018), maximum stone diameter (*P* = .001), preoperative hemoglobin (*P* = .005), diabetes (*P* = .05), eGFR < 30 mL/min/1.73 m^2^ (*P* = .0032), hypertension (*P* = .001), previous PCNL or pyelolithotomy (*P* = .0018), and severe hydronephrosis (*P* = .002) as risk factors for postoperative bleeding. The predictive model constructed based on these risk factors had an AUC of 0.73. In our study, the Random Forest model achieved a higher accuracy of 74.5%, with an AUC of 0.679. The slightly lower AUC in our model compared to Giorgio Mazzon et al may be due to differences in sample size. Further increasing the number of patients in the study for additional training is expected to enhance the model performance.

The application of machine learning algorithms is a future trend in the integration of medicine and technology, given their strong self-learning capability, continuous improvement, and optimization from data. While the models constructed in this study have yet to be widely applied in clinical settings, it represents a future direction. The developed software can directly connect to electronic medical record systems, incorporating real-time data for model training and performance enhancement. Urologists can receive timely postoperative predictions from machine learning models, providing valuable treatment references.

This study has some limitations. Firstly, the model architecture is based on single-center data, introducing potential bias. Secondly, compared to machine learning models in other fields, the sample size used for model training and testing in this study is still limited. Additionally, machine learning models are often referred to as black-box models, making it challenging to interpret specific statistical patterns, limiting their generalizability in the medical field.

## 5. Conclusion

We have successfully developed a relatively accurate predictive model for postoperative bleeding following PCNL based on machine learning algorithms. This model serves to forecast complications related to postoperative bleeding, providing valuable assistance to urologists in making timely and appropriate treatment decisions in the early stages.

## Author contributions

**Conceptualization:** Rui Meng.

**Data curation:** Rui Meng.

**Formal analysis:** Rui Meng.

**Funding acquisition:** Rui Meng.

**Project administration:** Weining Wang.

**Resources:** Weining Wang.

**Software:** Weining Wang, Zhipeng Zhai.

**Supervision:** Chao Zuo.

**Validation:** Zhipeng Zhai, Chao Zuo.

**Visualization:** Chao Zuo.

**Writing – original draft:** Chao Zuo.

**Writing – review & editing:** Chao Zuo.
